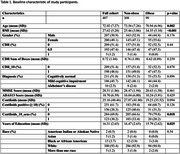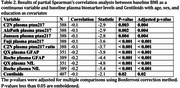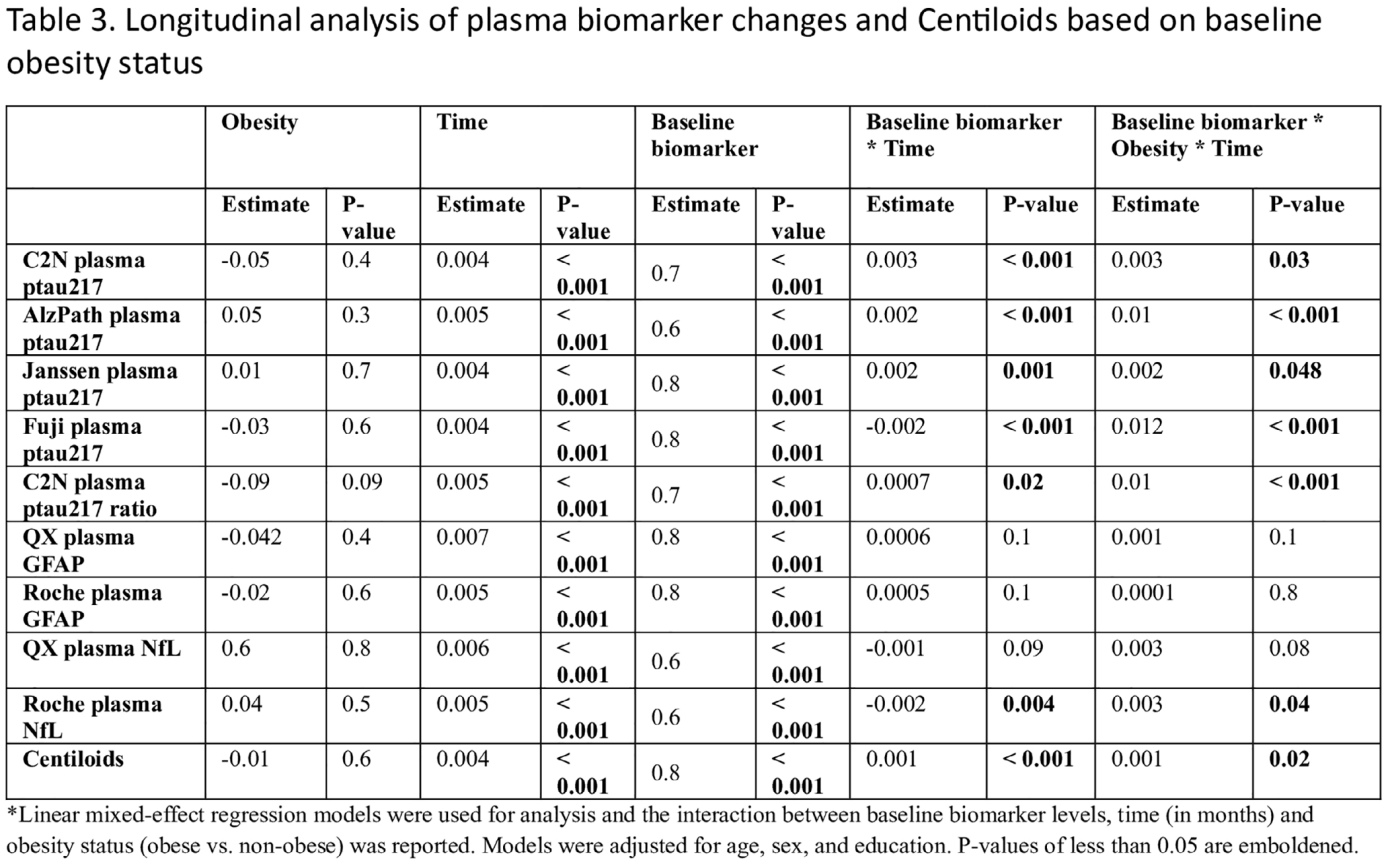# Trajectories of plasma biomarker levels and Amyloid Burden in Alzheimer's disease: A Longitudinal ADNI Study

**DOI:** 10.1002/alz70856_100022

**Published:** 2025-12-24

**Authors:** Soheil Mohammadi, Farzaneh Rahmani, Mahsa Dolatshahi, Suzanne E. Schindler, Cyrus A. Raji

**Affiliations:** ^1^ Mallinckrodt Institute of Radiology, Washington University in St. Louis, St. Louis, MO, USA; ^2^ Mallinckrodt Institute of Radiology, St. Louis, MO, USA; ^3^ Washington University in St. Louis, St. Louis, MO, USA

## Abstract

**Background:**

Obesity is a risk factor for Alzheimer's disease (AD), but its impact on blood biomarkers (BBMs) trajectories of AD remains unclear. This study investigates how obesity influences changes in plasma biomarkers and amyloid burden in AD.

**Method:**

BBM tests were performed on plasma samples from the Alzheimer's Disease Neuroimaging Initiative (ADNI) that were collected within six months of an amyloid positron emission tomography (PET) scan. Global amyloid burden was calculated as Centiloid values using (11)C‐Pittsburgh Compound‐B positron emission tomography. Spearman's partial correlation was used to assess the baseline cross‐sectional association between body mass index (BMI) and BBM levels, with age, sex, and years of education as covariates and adjusted for multiple comparisons. Linear mixed‐effects regression models were used to study three‐way interaction between baseline BBM, obesity (BMI>30), and time.

**Result:**

A total of 407 participants were included in the cohort, with a mean age of 72.92 years, 24.32% were classified as obese, 50.9% were male, and the mean Centiloid value was 25.16. At baseline, higher BMI was associated with lower amyloid burden as measured by PET as well as lower levels of plasma *p*‐tau217, %p‐tau217, GFAP and NFL. Obese participants had a faster increase in amyloid burden by PET and faster increases in plasma *p*‐tau217 and %p‐tau217 as compared to non‐obese individuals. However, obesity was not consistently associated with the rate of changes in NfL or GFAP.

**Conclusion:**

While obesity is mainly associated with lower baseline BBM levels, individuals with obesity tend to exhibit faster accumulation of amyloid burden by PET, as well as higher rates of changes in plasma *p*‐tau217 and %p‐tau217, compared to non‐obese individuals. The findings of this study not only offer valuable insights for interpreting BBM levels in obese versus non‐obese individuals but also provide insights on the effects of obesity on AD pathology over time.